# Practical Aquafeeds Incorporating Insect and Algae Meals Achieve Quality and Growth Standards Comparable to Traditional Feeds in Rainbow Trout (*Oncorhynchus mykiss*)

**DOI:** 10.3390/ani16071000

**Published:** 2026-03-24

**Authors:** Filippo Faccenda, Elia Ciani, Lorenzo Rossi, Gabriella Vale-Pereira, Giulia Secci, Jorge Dias, Luis E. C. Conceição

**Affiliations:** 1 Aquaculture and Hydrobiology Unit, Technology Transfer Centre, Fondazione Edmund Mach, 38098 San Michele all’Adige, Italy; elia.ciani@fmach.it (E.C.); lorenzo.rossi@fmach.it (L.R.); 2 Sparos Lda, 8700-221 Olhão, Portugal; aqi.gabriella@gmail.com (G.V.-P.); jorgedias@sparos.pt (J.D.); 3 Department of Agriculture, Food, Environment and Forestry (DAGRI), University of Firenze, Via delle Cascine 5, 50144 Firenze, Italy; giulia.secci@unifi.it

**Keywords:** rainbow trout, fillet quality, apparent digestibility, carbon footprint, black soldier fly, microalgae, macroalgae

## Abstract

This study assessed the effects of eco-efficient aquafeed formulations on the growth, body composition, nutrient retention, and flesh quality of rainbow trout (*Oncorhynchus mykiss*). Four extruded diets were tested: a conventional control (Ctrl) and three eco-efficient formulations (No-PAP, PAP, and Mix) combining single-cell ingredients, insect meal, selected plant proteins, and aquaculture by-products, with long-chain omega-3 fatty acids (DHA and EPA) supplied primarily from microalgae. Rather than isolating single-ingredient replacements, the objective was to evaluate the efficacy of complex, industry-feasible formulations designed for practical application. These experimental feeds were administered to 800 trout (initial body weight 63 g), distributed across four replicates per diet. After a 97-day trial, results showed no significant differences in growth performance (final weight, weight gain, and relative growth rate) between diets. Feed conversion ratios (~0.78) were within expected ranges for rainbow trout under these conditions. Fillet texture and composition were similar across all groups; however, trout fed the No-PAP diet developed a significantly more yellow tint in fillet color, likely attributed to xanthophyll pigments from plant- and algae-based ingredients. The environmental impact of the diets was driven by specific ingredient choices, as evidenced by a carbon footprint analysis ranging from 1.39 kg CO_2_ eq. (PAP diet) to 2.36 kg CO_2_ eq. (Ctrl diet). These findings demonstrate that the three alternative formulations matched the efficacy of conventional feed, offering a sustainable option for trout aquafeed production.

## Simple Summary

Global food production places significant pressure on natural resources and remains a primary driver of climate change. While aquaculture is generally more efficient than terrestrial livestock, its reliance on marine-derived fishmeal and fish oil is a persistent challenge. This study evaluated alternative and eco-friendly feed formulations for rainbow trout, substituting traditional ingredients with a blend of insect, microbial, and yeast proteins, alongside algae and sustainable by-products. The results demonstrate that these alternative diets achieve growth rates and fillet quality comparable to conventional feeds. By proving that innovative ingredients can successfully replace fishmeal, this research supports the advancement of sustainable aquaculture, helping to alleviate pressure on marine ecosystems and supporting food security.

## 1. Introduction

The food system generates considerable pressure on natural resources and the environment, resulting in one of the major drivers of climate change. In particular, greenhouse gas (GHG) emissions, deforestation, land occupation, freshwater consumption, and eutrophication are commonly associated with food production [1,2]. At the same time, the food production chain still fails to meet global nutrition needs, with 1 in 9 people lacking sufficient food [3]. Therefore, the current and future challenge of the food sector is to produce more food while, at the same time, improving the environmental performance of the entire supply chain. In this context, fish and other aquatic foods (so-called ‘blue foods’) present an opportunity towards sustainable nutrition with lower environmental burdens [1]. According to the most recent FAO estimates, in 2022, global production from aquaculture (94.4 million tonnes) exceeded fishing production (91.0 million tonnes) [4]. Moreover, aquaculture has become more integrated into the global food system, with rapid transformations in feed formulations, production system technologies, farm management, and value chains [5]. The environmental impact of aquaculture is lower compared to other terrestrial animal productions, in particular: 87% smaller carbon footprints than beef, 49% less land consumption than poultry, and 84% less freshwater use than pigs [2]. In absolute terms, unfed aquaculture, i.e., farmed macroalgae and bivalves, generates the lowest carbon emissions, while fed aquaculture, i.e., fish and shrimp, results in higher carbon footprints [1]. This is due to the production and consumption of fish and shrimp feeds, which are responsible for more than 70% of emissions [1]. In this context, the contribution of salmonids aquaculture resulted in 10,102 thousand tons of CO_2_ eq., which is approximately 4% of global aquaculture impact [6]. Among salmonids, rainbow trout (*Oncorhynchus mykiss*) is one of the most important farmed species worldwide. The global production in 2023 was roughly 1.1 million tonnes, with Turkey and the Islamic Republic of Iran representing the largest producers (19.9 and 19.5% of global production, respectively) [7]. The European Union contributed 0.17 million tonnes of global production, mostly obtained by Italy (20%), France (16.8%), Denmark (14.4%), and Poland (10.7%) [8].

One of the principal criticisms of aquaculture concerns the use of marine resources, particularly forage fish, for the production of fishmeal (FM) and fish oil (FO). In 2022, the aquaculture sector consumed approximately 86% of global FM and 72% of global FO production [4]. Over the past two decades, the sector has considerably enhanced its efficiency [5]; while production has surged—surpassing capture fisheries for the first time in 2022—the use of wild-caught marine ingredients has stabilized. This progress is largely due to improved feed conversion ratios (FCRs), the growth of omnivorous species, and the adoption of a circular bio-economy framework [9]. A key sustainability driver is the valorization of marine side-streams: in 2022, 34% of global FM and 53% of global FO were produced from fish-processing wastes and by-products [4]. However, as the industry faces a projected requirement of an additional 37.4 million tonnes of fish and shrimp feeds to meet near-term demand, relying on a few traditional ingredients is no longer viable [10]. The fish and shrimp feed industry is transitioning toward a “multi-source” strategy that combines traditional plant-based ingredients (soybean, corn, and rapeseed) with emerging resources. These include marine low-trophic species (mesopelagic fish, zooplankton, and macroalgae), novel microbial ingredients (bacteria, yeast, and microalgae), insects (black soldier fly and yellow mealworm), and various terrestrial animal by-products (poultry meal, meat and bone meal, blood meal, and hydrolyzed feather meal) and fisheries by-products (trimmings and blood) [9,10,11]. While terrestrial plant ingredients have mitigated FM reliance during the last decade, they face limitations regarding land use and water competition. Consequently, FM and FO remain the “gold standard” for carnivorous species like salmonids, but their role is evolving from bulk staples to strategic, high-value specialty ingredients used to ensure the final product’s nutritional quality for human consumers [4,10]. Despite the diversification of aquafeeds, the transition toward alternative proteins introduces a significant “sustainability paradox.” As noted by Hua et al. [10] and Eroldoğan et al. [9], replacing marine resources with terrestrial alternatives often merely shifts environmental pressure to land and water resources, while introducing anti-nutritional factors and unbalanced amino acid profiles that can compromise fish gut health. Furthermore, FAO [4] highlights emerging food safety concerns, such as mycotoxin contamination in crops and heavy metal bioaccumulation in low-trophic marine organisms. Consequently, the industry must balance environmental sustainability against cost-efficiency, year-round availability, and palatability, while ensuring the final fillet composition maintains its nutritional value for human consumers [2,12].

To address these multifaceted challenges, the present study moves beyond traditional single-ingredient substitution. Instead, we evaluate a “system-level” formulation strategy for rainbow trout (*Oncorhynchus mykiss*). This approach utilizes a diverse “basket” of emerging ingredients, including insect, microbial, and yeast proteins alongside macro- and microalgae, to create functionally balanced, eco-efficient diets. By conducting a comprehensive multi-criteria assessment—linking growth performance and nutrient use efficiency to fillet quality and environmental carbon footprints—this research demonstrates how innovative, practical formulations can effectively replace fishmeal and fish oil while mitigating the ecological impacts of modern aquaculture.

## 2. Materials and Methods

### 2.1. Diet Composition

Four floating diets (Table 1), formulated according to the known nutritional requirements for rainbow trout [13], were manufactured by extrusion at SPAROS Lda. (Olhão, Portugal). Rather than a traditional single-factor replacement, the formulation strategy followed a basket-based rationale designed to validate functional nutritional equivalence across three contrasting—but nutritionally adequate—eco-efficient ingredient combinations. The four diets included: (i) a control diet (Ctrl), reflecting common commercial practices with 20% fishmeal (FM) and a high inclusion of soy protein concentrate (SPC); (ii) a diet without terrestrial processed animal proteins (No-PAP); (iii) a diet incorporating high level of processed animal proteins (PAP); and (iv) a mixed diet (Mix) combining elements of both alternative baskets. The No-PAP diet emphasized plant proteins, single-cell proteins (SCPs), and micro-/macroalgae, intentionally excluding terrestrial PAPs to enhance circularity while addressing negative consumer perceptions sometimes associated with PAP use. In contrast, the PAP diet prioritized processed animal proteins—including poultry meal, feather meal hydrolysate, and porcine hemoglobin—supplemented with insect and microbial meals, in order to reduce FM while meeting limiting essential amino acid and phosphorus constraints and improving resource circularity. The Mix diet blended the strengths of both baskets, incorporating combinations of insect meal (*Hermetia illucens*), microbial protein meal, yeast protein meal, plant proteins (e.g., wheat gluten and pea protein concentrate), and selected PAP sources. Across all alternative diets, a blend of brewer’s yeast and macroalgae was included, and the No-PAP and Mix diets also incorporated microalgae meals from *Spirulina* sp. and *Chlorella* sp. Salmon oil derived from aquaculture by-products replaced marine fish oil in every alternative formulation, while DHA-rich microalgae (*Schizochytrium* sp.) ensured the required long-chain n-3 PUFA levels. Inclusion levels in all diets were determined through least-cost formulation under strict nutritional constraints (essential amino acids, digestible protein and energy, and EPA + DHA targets). This approach ensured that comparisons were based on metabolic functionality while simultaneously accounting for ingredient availability and industrial feasibility. Complete information on the ingredients, manufacturing protocol, and the amino acidic, fatty acid, mineral, and vitamin composition is available in Supplementary File S1.

### 2.2. Dietary Trials

The trial was conducted at the experimental fish plant of the Edmund Mach Foundation (FEM) in San Michele all’Adige (Italy). All fish handling procedures adhered to the EU legal framework for the protection of animals used for scientific purposes (Directive 2010/63/EU) and were approved by the Animal Welfare Committee (n. 9750/2019).

The experimental pool of 800 rainbow trout (*Oncorhynchus mykiss*), with an average initial body weight (IBW) of 63 ± 1.31 g, was randomly allocated to 16 tanks (50 animals per tank approx. 700 L of water). These tanks were a subset of the FEM indoor research plant that utilized well water in a flow-through rearing system. During the test, the water temperature was 12.7 ± 0.2 °C, and the dissolved oxygen level was 8.4 ± 0.6 mg/L (Supplementary Figure S1). The tanks experienced a natural photoperiod at the location coordinates (46°11′30.3″ N 11°08′05.3″ E). Animals were acclimatized for a week prior to the experiment start. Four tanks were assigned to each of the four experimental groups (diets). Animals were hand-fed to apparent satiation in two meals per day (09:00–14:00), six days per week, for 97 days. The amount of feed administered and uneaten was recorded daily at the tank level. At the beginning of the trial and again on day 97, individual fish were anesthetized for weighing and measuring. Additionally, a bulk weight for each tank was recorded on day 48, marking the midpoint of the trial. At the start, 10 fish from the initial stock were sampled and stored at −20 °C for later whole-body composition analysis. Following 97 days of feeding, 6 fish from each tank were collected for carcass and flesh quality analyses. Fish were euthanized via anesthetic overdose (400 mg/L MS-222).

### 2.3. Growth Performance and Feed Efficiency

Growth performance and feed efficiency were evaluated through the following parameters: mean initial body weight (IBW), mean final body weight (FBW), relative growth rate (RGR), feed conversion ratio (FCR), feed intake (FI), protein efficiency ratio (PER), and retention (Ret). Such values were calculated as follows:

(1) $$\mathrm{IBW}\;(g)\;=\;\frac{ITB}{n}$$

(2) $$\mathrm{FBW}\;(g)=\frac{FTB}{n}$$

(3) $$\mathrm{RGR}\;(\%\;\mathrm{BW}/\mathrm{day})=\left(e^{\frac{Ln\;FBW-Ln\;IBW}{d}}-1\right)*100$$

(4) $$\mathrm{FCR}=\frac{CFI}{WG}$$

(5) $$\mathrm{FI}\;(\%\;\mathrm{BW}/\mathrm{day})=\left(\frac{CFI}{\frac{ITB+FTB}{2}*m}\right)*100$$

(6) $$\mathrm{PER}=\frac{WWG}{CPI}$$

(7) $$\mathrm{Ret}\;(\%)=\left(\frac{\left(FBW*NFF\right)-(IBW*NIF)}{NI}\right)*100$$

where Initial Total Biomass (ITB); Final Total Biomass (FTB); number of animals (n); days of trial (d); number of meals (m); Crude Feed Intake (CFI); Weight Gain (WG) corrected for mortalities and sampled animals; Wet Weight Gain (WWG); Crude Protein Intake (CPI); Nutrient content of final fish (NFF); Nutrient content of initial fish (NIF); and Nutrient Intake (NI).

### 2.4. Composition Analysis in Fish, Feed, and Feces

Analyses of diets, whole fish, and feces were performed in analytical duplicates following the methodologies outlined by AOAC (2006) [14]. Dry matter content was assessed by drying samples at 105 °C for 24 h. Total ash was measured by combustion in a muffle furnace at 550 °C for 6 h. Crude protein (N × 6.25) was determined using a flash combustion technique, followed by gas chromatographic separation and thermal conductivity detection with a Leco N Analyzer (Model FP-528, Leco Corporation, St Joseph, MI, USA). Crude lipid was measured after acid hydrolysis using dichloromethane extraction (40–60 °C) with a Soxtec™ 2055 Fat Extraction System (Foss Analitics, Hilleroed, Denmark). Gross energy was evaluated in an adiabatic bomb calorimeter (model C 2000 Basic, IKA-Werke GmbH & Co, Staufen, Germany). The concentration of yttrium oxide (Y_2_O_3_) in both feed and fecal samples was measured using atomic absorption spectrometry (Model SpectrAA 220 FS, Varian Inc., Palo Alto, CA, USA) [15].

### 2.5. Apparent Digestibility

Feces were collected on day 97 to determine the apparent digestibility coefficients (ADC [13]) of the diets using the indirect method [16]. Throughout the experimental period, all fish diets included Y_2_O_3_ at a concentration of 0.02% as a non-reactive marker. Fecal samples were stripped from anesthetized fish [17], pooled by tank in plastic containers, and two 100 g subsamples were frozen at −20 °C for later laboratory analysis. ADC was calculated as follows:

(8) $$\mathrm{ADC}\;(\%)\;=\;\left(1-\frac{\%\;MD}{\%MF}*\frac{\%\;TNF}{TND}\right)*100$$

where marker diet (MD); marker feces (MF); target nutrient feces (TNF); and target nutrient diet (TND).

### 2.6. Fish and Fillet Quality Analysis

The marketable quality of the fish and fillet was evaluated based on body indexes, along with texture profile analysis (TPA), color, and chemical composition (see Section 2.4) of the fillet.

#### 2.6.1. Body Indexes

Six fish from each tank (24 fish per diet) were collected at the end of the trial. After slaughter, the fish were stored at 4 °C for 24 h. The following day, the fish were eviscerated, and the carcass, whole viscera, liver, and mesenteric fat were weighed. Carcass yield (CY; %), hepato-somatic index (HSI; %), viscero-somatic index (VSI; %) and mesenteric fat index (MFI; %) were calculated as follows:

(9) $$\mathrm{CY}\;(\%)\;=\;\left(\frac{BW-vW}{BW}\right)*100$$

(10) $$\mathrm{HSI}\;(\%)=\left(\frac{lW}{BW}\right)*100$$

(11) $$\mathrm{VSI}\;(\%)=\left(\frac{vW}{BW}\right)*100$$

(12) $$\mathrm{MFI}\;(\%)=\left(\frac{mfW}{BW}\right)*100$$

where body Weight (BW); visceral Weight (vW); liver weight (lW); and mesenteric fat weight (mfW). All weights were measured in grams. Fish were filleted afterwards.

#### 2.6.2. Texture Profile Analysis (TPA)

Textural properties were measured on a muscle sample (4 × 4 cm) taken from the epiaxial region of the right fillet. The TPA was performed using a Zwick Roell^®^ 109 texturometer (ZwickRoell GmbH, Ulm, Germany), equipped with a 1 kN load cell and a cylindrical probe (10 mm), operated via Text Expert II software (version 3.0). Two consecutive compression cycles, with a 5 s interval between them, were applied to 50% of the sample’s total deformation at a crosshead speed of 100 mm/min [18,19]. The following parameters were determined as described in Veland at al., 1999 [19]: (1) Hardness (Newton, N): the peak force that occurs during the first compression; (2) Cohesiveness: indicates how well the product withstands a second deformation relative to its resistance under the first deformation $\left(\frac{Area\;2}{Area\;1}\right)$; (3) Gumminess (N): the energy required to masticate a semi-solid food product to the point of swallowing, calculated as Hardness × Cohesiveness; (4) Resilience (N*mm): indicates how well a product regains its original height after compression, calculated by dividing the upstroke energy of the first compression by the downstroke energy of the first compression $\left(\frac{Area\;4}{Area\;3}\right)$; and (5) Adhesiveness (N*mm): the work required to pull a probe away from a sample after it has been compressed.

#### 2.6.3. Colorimetric Analysis

**Figure 1:**
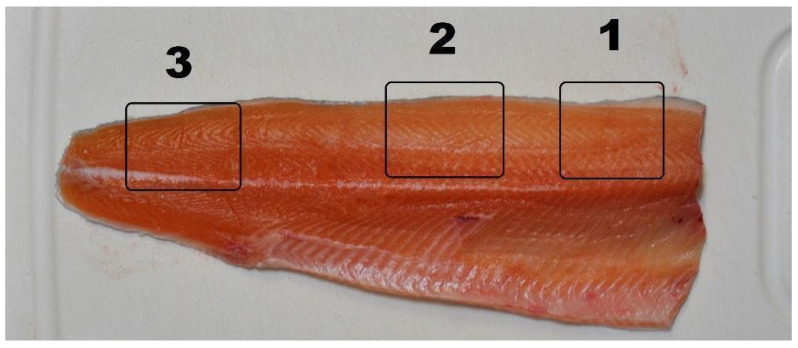
Sampling points for color analysis on the left fillet (1. cranial, 2. dorsal, and 3. caudal section). Picture modified from Vale Pereira et al., 2023 [20].

The color of the meat was measured in triplicate on the cranial, dorsal, and caudal sections of the left fillet (Figure 1).

A Konica Minolta CR-400 colorimeter (Konica Minolta, Tokyo, Japan) was used for color measurement according to the CIE-Lab system [21]. In this color space, L* indicates lightness (from 0: completely black to 100: completely white), while a* and b* refer to chromaticity coordinates (a*: red-green index; b*: yellow-blue index). Chroma [C—an expression of color intensity (saturation)], hue (h—the angular measurement of the tint, expressed in radians), and Entire Color Index (ECI [22]) were calculated as follows:

C = (a*^2^ + b*^2^)^0.5^ (13)

h = arctan(b*/a*)(14)

(15) $$\mathrm{ECI}=C_{i}*\cos(h_{i}-\underline{\bar{h}})$$

where $\bar{h}$ is the average hue, and Ci-hi are the chromaticity and hue values of each measurement. The ECI formula was applied to consider hue and chromaticity as a single variable, allowing for a unique identification of the color of the fillet.

### 2.7. Environmental Impact Assessment of Diets

The environmental impact assessment was conducted considering the impact on global warming potential (i.e., GHG emissions as kg CO_2_ equivalent), as it represents one of the most important impact categories associated with feed production in aquaculture [23]. The impact was calculated considering two functional units: (i) 1 kg of feed and (ii) 1 kg of fish produced, considering the calculated FCR per treatment. The impact considered refers to the production of individual feed ingredients at the plant level, i.e., transportation was not considered. The references used for each ingredient are listed in Table 2.

### 2.8. Statistical Analysis

Statistical analyses were performed using R Statistical Software (v4.1.0 [37]) and STATISTICA (v14.0.1.25, StatSoft GmbH, Hamburg, Germany). Data were visually tested for normal distribution and homoscedasticity by histogram and scatter plot. Statistically significant (*p* < 0.05) differences between groups were investigated by analysis of variance (ANOVA or ANCOVA), followed by Tukey pairwise multiple comparison (Bonferroni adjustment). The number of replicates included in the statistical models varied depending on the variable analyzed. For variables calculated at the tank level (e.g., feed conversion ratio, FCR; relative growth rate, RGR), the tank was considered the experimental unit (*n* = 4 tanks per diet). Fish biometric parameters (e.g., body weight, total length, and condition factor, K) were measured on all individual fish (*n* = 200 fish per dietary treatment). Fillet coloration, texture, carcass yield (CY), viscero-somatic index (VSI), and hepatosomatic index (HSI) were evaluated on 24 fish per dietary treatment, whereas visceral fat index (VFI) was measured on 21 fish per dietary treatment. Values expressed as percentages were arcsine square root transformed to meet test assumptions. Quality of the models was evaluated by plotting of residuals (e.g., Q-Q plot, residuals vs. fitted).

## 3. Results

### 3.1. Growth Performances

**Figure 2:**
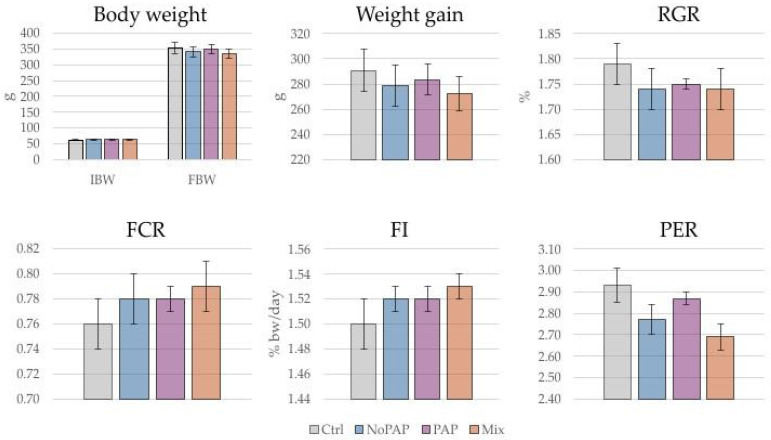
Growth performance parameters (mean ± SD) for fish fed the different experimental diets [(i) Control diet (Ctrl); (ii) diet without Processed Animal Proteins (No-PAP); (iii) diet with Processed Animal Proteins (PAP); and (iv) mixed diet (Mix)]. Relative Growth Rate (RGR); Feed Conversion Ratio (FCR); Feed Intake (FI); Protein Efficiency Ratio (PER). Note that body weight was measured in all individual fish (*n* = 200 per group), while other parameters were measured at the tank level (*n* = 4 per group).

During the 97-day trial, the trout accepted all diets readily, with a daily feed intake ranging from 2.4% to 1.4% of the estimated biomass. No discernible differences in feed consumption or appetite were observed between diets or replicates. Environmental conditions remained within the optimal range for rainbow trout throughout the experimental period. At the conclusion of the trial, fish fed the alternative formulations exhibited growth performances comparable to the control group (Figure 2). No statistically significant differences (*p* > 0.05) were detected between groups for any of the analyzed parameters. Mortality was negligible, with only one fish death recorded in the PAP treatment over the entire period. Final body weight (FBW) ranged between 335 ± 14.3 (Mix) and 353 ± 17.5 g (Ctrl), representing a 5.4-fold increase from the initial body weight (IBW). Across all dietary groups, the average relative growth rate (RGR) was approximately 1.76% day^−1^, the feed conversion ratio (FCR) was approximately 0.78, and the feed intake (FI) averaged 1.52% BW/day. The protein efficiency ratio (PER) ranged from 2.69 (Mix) to 2.93 (Ctrl).

### 3.2. Marketable Traits

**Figure 3:**
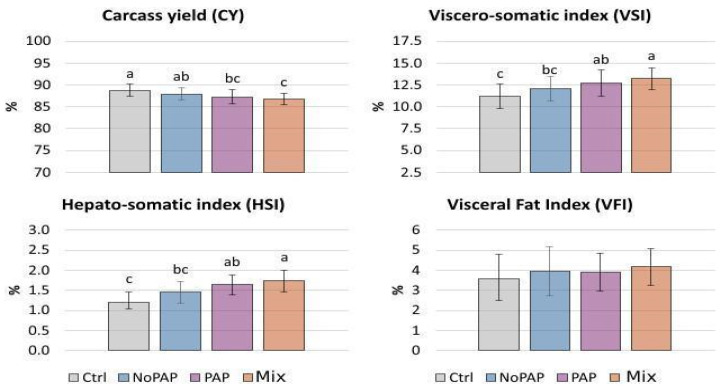
Morphological indexes of fish exposed to the different experimental diets at the end of the trial [(i) Control diet (Ctrl); (ii) diet without Processed Animal Proteins (No-PAP); (iii) diet with Processed Animal Proteins (PAP); and (iv) mixed diet (Mix)]. Different letters denote statistically significant differences (*p* < 0.05) between groups. mean % ± SD; CY-VSI-HSI *n* = 24 per group; VFI *n* = 21 per group.

Morphological differences were detected between fish exposed to different experimental diets (Figure 3). The Ctrl diet reached the best carcass yield with 88.78%, followed by No-PAP (87.92%), PAP (87.29%), and Mix diet (86.77%). Carcass yield (CY) is directly affected by viscera weight, with the viscero-somatic index (VSI) showing an inverse relationship to CY. The Mix diet resulted in the highest VSI (13.23%), followed by PAP (12.71%) and No-PAP (12.08%), and the lowest was observed in the Ctrl group (11.22%). A similar significant trend was observed in the hepatosomatic index (HSI), with a proportionally larger liver size in fish fed the Mix diet compared to other groups. No statistical differences were detected in visceral fat across diets.

### 3.3. Nutrient Retention

**Figure 4:**
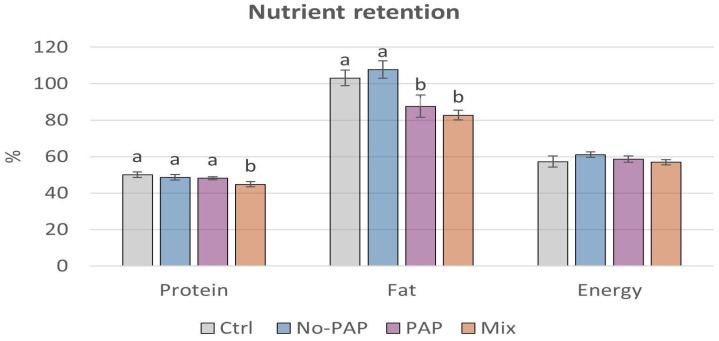
Nutrient retention (mean % ± SD) for fish fed different dietary treatments [(i) Control diet (Ctrl); (ii) diet without Processed Animal Proteins (No-PAP); (iii) diet with Processed Animal Proteins (PAP); and (iv) mixed diet (Mix)]. Different letters indicate statistically significant differences between groups (*p* < 0.05); *n* = 4 per group.

Fish fed the Mix diet showed lower protein retention compared to the other groups (Figure 4). Fish fed the PAP and Mix diets exhibited lower fat retention compared to the other groups. No differences in energy retention were detected among the experimental diets.

### 3.4. Apparent Digestibility (Feces)

**Figure 5:**
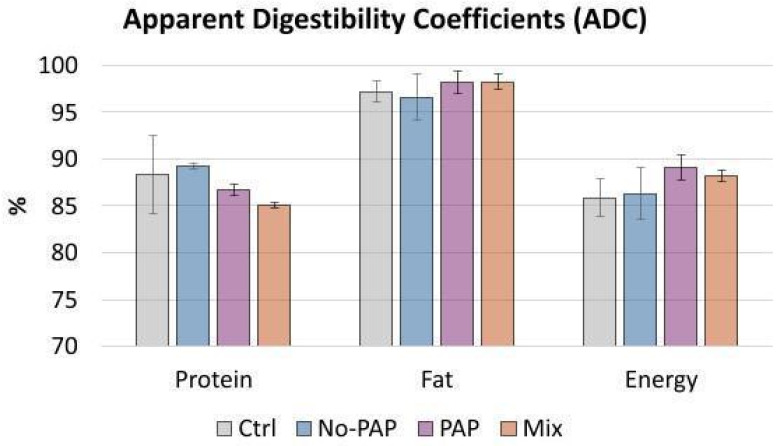
ADC (mean % ± SD) for fish fed different dietary treatments [(i) Control diet (Ctrl); (ii) diet without Processed Animal Proteins (No-PAP); (iii) diet with Processed Animal Proteins (PAP); and (iv) mixed diet (Mix); *n* = 4 per group.

Dietary treatments had no significant effect (*p* > 0.05) on ADC values (Figure 5). Mean values at the end of the trial were as follows: protein 87.6%, fat 97.5%, and energy 87.4%.

### 3.5. Characterization of Fillet Quality

#### 3.5.1. Fillet Texture

Dietary treatments did not significantly affect (*p* > 0.05) fillet texture, as assessed by the determined parameters (Table 3).

#### 3.5.2. Fillet Color

**Figure 6:**
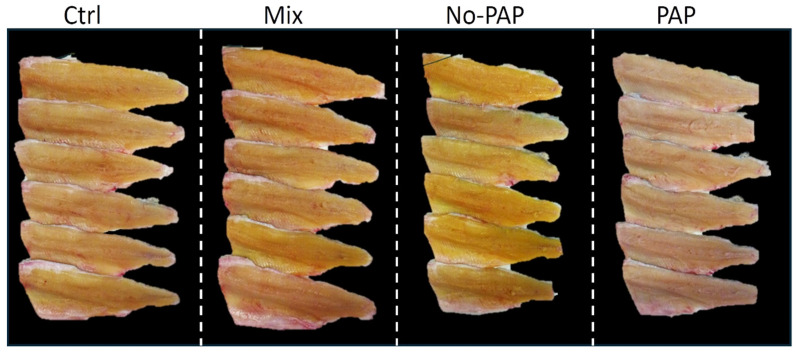
Image showing six representative fillets per diet: (i) Control diet (Ctrl); (ii) diet without Processed Animal Proteins (No-PAP); (iii) diet with Processed Animal Proteins (PAP); and (iv) mixed diet (Mix).

Differences in fillet coloration among dietary treatments were apparent upon visual inspection (Figure 6). Fillets from fish fed the No-PAP diet displayed a distinct yellow hue, whereas those from the PAP diet appeared noticeably paler. Fillets from the Ctrl and Mix diets exhibited intermediate coloration.

**Figure 7:**
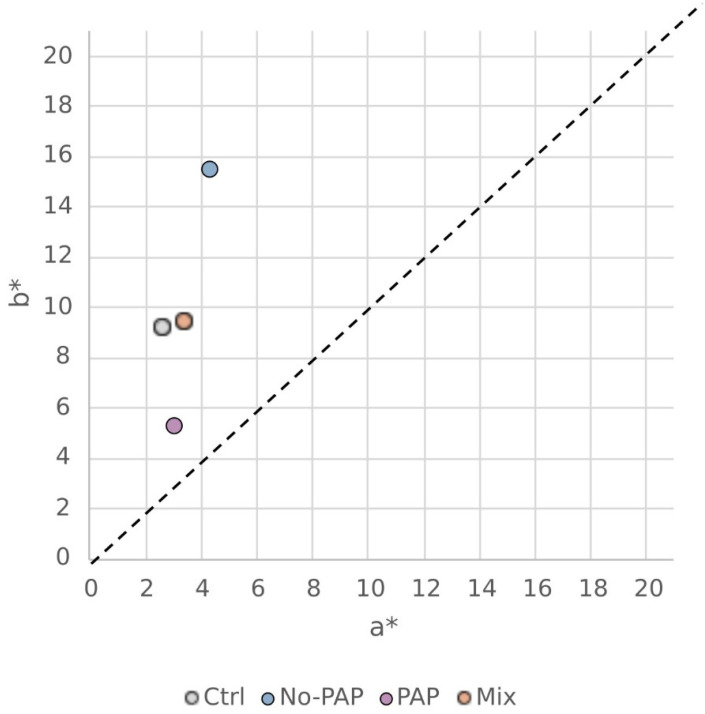
Scatter plot showing mean values for a* (redness index) and b* (yellowness index) across four dietary groups: (i) control diet (Ctrl), (ii) diet without processed animal proteins (No-PAP), (iii) diet with processed animal proteins (PAP), and (iv) mixed diet (Mix); *n* = 216 reads per group (24 fish with 3 fillet-areas each and 3 reads per area).

These visual observations were confirmed by colorimetric analysis (Figure 7), which revealed that the PAP and No-PAP diets produced the greatest divergence in color parameters, while the Ctrl and Mix diets showed comparable, intermediate values.

Statistical analysis confirmed that these differences were significant (Table 4). Using CIELab color parameters, the lightness (L*) of PAP fillets was significantly higher than in the other three diets, indicating a paler appearance. Both Mix and Ctrl diets showed similar L* values, while No-PAP fillets had the lowest L*. The No-PAP diet also led to the highest values of red (a*) and yellow (b*) indices, with Mix and Ctrl diets showing intermediate levels, and PAP fillets having the lowest values. In the LCh* color space, No-PAP fillets exhibited the highest chroma (C), followed by Mix and Ctrl groups, with PAP fillets showing the dullest intensity. For hue, No-PAP and Ctrl resulted in similar hues, both statistically distinct from the Mix and PAP diets, with the PAP diet having the smallest hue angle. The Ctrl and PAP diets yield similar ECI values without statistically significant differences, while both differ significantly from the Mix diet and the No-PAP diet.

#### 3.5.3. Fillet Composition

Dietary treatments had no significant effect (*p* > 0.05) on fish fillet composition (Table 5). Mean values at the end of the trial were as follows: moisture = 65.8%, ash = 1.6%, protein = 16.3%, fat = 12.6%, and energy = 9.6%.

### 3.6. Environmental Impact of Diets

The environmental impact of the diets (Table 6) ranged from 1.24 to 1.91 kg CO_2_ eq. for PAP and No-PAP, respectively. Considering the impact of fish produced, the results are similar, with PAP treatment showing the lowest value (0.97 kg CO_2_ eq.) and No-PAP treatment showing the highest (1.49 kg CO_2_ eq.). The contribution analysis (Table 6) showed that the impact of the Ctrl diet is mostly associated with plant-based ingredients, of which wheat gluten meal (16% of the overall impact), SPC (14%), and rapeseed oil (14%) are the most impactful ones. Considering the alternative diets, the impacts in No-PAP and Mix treatments are mostly related to the use of microalgae meal obtained from *Spirulina* sp., which accounted for 19% and 21% of the overall impact, respectively. Also, microbial protein meal (13% of the overall impact) and insect meal (10%) contribute significantly to the Mix diet impact. In the PAP diet, the lipid ingredients, such as salmon oil from by-products and DHA-rich oil obtained from microalgae, represent the highest contributors to GHG emissions with 12% and 11% of the overall impact, respectively.

## 4. Discussion

The development of sustainable aquafeeds represents a critical research frontier, aimed at decoupling aquaculture from its reliance on finite marine resources such as fish meal (FM) and fish oil (FO). Furthermore, reducing the utilization of traditional terrestrial ingredients, like soy and wheat, is essential to alleviate pressure on natural ecosystems and mitigate the industry’s contribution to climate change. Consequently, novel formulations prioritizing eco-efficiency and circular economy principles are increasingly vital for the sector’s sustainable growth. Building upon these principles, the present study establishes a robust framework to evaluate practical, industry-feasible formulations that translate theoretical sustainability into ready-to-use applications. A core feature of this research is the integration of a multi-criteria evaluation that extends beyond standard growth performance to include apparent digestibility coefficients (ADC), environmental impacts (carbon footprint), fillet quality, and consumer acceptance (texture, color, and composition). Our findings demonstrate that these ingredient “baskets” can match the performance of conventional controls (Ctrl) while simultaneously revealing critical trade-offs, such as those identified in the carbon footprint and pigmentation analyses.

While a traditional isonitrogenous and isolipidic template is often used to isolate single-ingredient effects, our experimental design prioritized functional nutritional balancing to evaluate the efficacy of entire formulation systems. Although the experimental diets were not strictly isonitrogenous or isolipidic, the minor variations in crude protein and lipid levels reflect realistic manufacturing constraints, ingredient availability, and the intrinsic goals of each formulation basket. Several safeguards were implemented to ensure scientific validity and comparability across treatments. First, all diets met or exceeded NRC/IAFFD nutrient requirements for rainbow trout at the tested life stage. Second, gross energy and protein-to-energy ratios remained within narrow, nutritionally appropriate ranges, supporting equivalent metabolic conditions across groups. Third, all performance endpoints—including final body weight (FBW), weight gain (WG), feed conversion ratio (FCR), and protein efficiency ratio (PER)—were generated under identical, tightly controlled rearing conditions. Fourth, the high and comparable ADCs across treatments confirm similar nutrient availability, further validating functional equivalence despite minor compositional differences. This pragmatic formulation approach—balancing for nutrient functionality rather than forced isonitrogenous templates—has been widely adopted in previous GAIN project studies on gilthead seabream, turbot, and various salmonids [20,38,39,40,41,42]. The consistency of the FCR and PER across all experimental groups provides strong physiological evidence that the diets were effectively balanced for nutrient and energy utilization; if a significant nutritional deficit had existed due to the reduction of FM, it would have been reflected in diminished growth performance, which was not observed. The consistency across species and trials supports the validity of this comparative design as both nutritionally sound and industrially relevant.

Regarding feeding management, the use of manual feeding was standardized to ensure robustness. Fish were hand-fed to apparent satiation twice daily, six days per week, with daily feed intake (FI) precisely recorded at the tank level. This protocol ensures that feeding is applied uniformly across treatments and that any intake variation is captured in the calculation of FCR and PER. Consequently, the evaluation is based on actual recorded consumption rather than assumed amounts. This methodology is consistent with validated protocols used in previous large-scale trials [20,38,39,40,41,42], where hand-feeding to satiation has reliably supported unbiased diet comparisons. By adopting this comprehensive approach, this study provides actionable insights to optimize sustainability within both current and predictable regulatory frameworks.

### 4.1. Growth Performance and Feed Utilization

A key finding of the present study was that fish fed with different diet formulations showed comparable growth performances. After a three-month trial, no differences were detected in final body weight, growth rate, feed conversion ratio, feed intake, or protein efficiency ratio (PER). This suggests that these novel formulations can effectively match the efficacy of traditional feeds in terms of supporting robust growth in rainbow trout. The consistent growth performance data were supported by the calculated ADCs, which were mostly unaffected across all diets. This indicates that the trout maintained general efficiency in nutrient absorption from the alternative diet formulations, even if some differences in specific nutrient retention existed. This stability contrasts with another study that found that protein ADC and retention in rainbow trout could be slightly impacted by PAP-based formulations [20].

These results align with those of similar studies across important aquaculture species, indicating a general trend toward the successful replacement of traditional feed formulations with eco-efficient alternatives without compromising growth or feed conversion. This trend has been observed in gilthead seabream [38,43], turbot [39,40], European seabass [41], and other rainbow trout research [20,44].

However, while overall growth might be maintained, feed efficiency parameters like FCR and PER can show nuanced, species-specific differences, depending on the diet composition. For example, in a study with gilthead seabream [42] using alternative diets similar in concept to the present study, three experimental diets supported comparable growth rates. Yet, the No-PAP diet actually demonstrated the best FCR and PER among the experimental groups, suggesting it promoted improved nutrient absorption and/or utilization in seabream. Biomarker analysis in that study also points to improved physiological condition (lower oxidative/inflammatory signatures) and showed adaptive hepatic lipogenesis without pathological outcomes—patterns compatible with EAA adequacy and microbiome-mediated SCFA fueling of lipid metabolism [42,45]. These host–microbiome and biomarker findings provide a plausible explanation for the favorable FCR/PER observed with balanced No-PAP baskets.

For turbot [40], juveniles fed a PAP diet showed a significantly reduced FCR compared to the control. For market-sized turbot [39], FCR was significantly higher, worse in one PAP and two No-PAP groups compared to the Ctrl group, and PER was consistently lower in two PAP groups. The same study also observed that No-PAP formulations allowed for better utilization than PAP formulations, irrespective of the FM replacement level. In European seabass [41], No-PAP and PAP diets showed a significantly lower relative growth rate compared to the Ctrl, though PER values were generally within the expected range, with Ctrl and NO-PAP diets showing higher PERs.

Considering the important role of diet formulation and how single ingredients can affect nutrient utilization across different species [20,39,40,42,44], the consistent and efficient performance of rainbow trout in the present study makes these results highly encouraging for the adoption of sustainable aquafeeds. Across species, No-PAP baskets can deliver favorable physiology and efficiency signatures (microbiome/biomarker-supported), while PAP baskets maintain growth with outcomes contingent on digestibility and co-ingredient choice. When functional nutrient adequacy (EAA, digestible protein/energy, and EPA + DHA) is respected, eco-efficient baskets provide nutritionally sound and industrially feasible alternatives to conventional feeds [20,39,40,41,42,44,45]. Trout and gilthead seabream have a very good tolerance to alternative well-balanced formulations, with adaptive metabolic responses to nutrient composition, while turbot and seabass may have only a moderate capacity for such adaptation.

### 4.2. Fillet Quality

The results of the present study indicated similar fillet texture, protein, lipid, and energy content across all dietary groups. However, a notable qualitative difference emerged in trout fillet color, a key determinant of marketability.

A characteristic pale grey or light pink color is often observed in the flesh of farmed rainbow trout that are not supplemented with carotenoids. Indeed, it is well established that salmonid flesh color is derived from dietary pigments, especially carotenoids, since these fish cannot synthesize these molecules de novo. Carotenoids are a broad family of xanthophylls and carotenes that impart a range of colors from yellow to pink and orange. Specifically, astaxanthin and canthaxanthin are known to produce the orange or pink hues desirable in salmonid flesh, due to their positive impact on consumer appeal [46]. Pigment metabolism, absorption, and deposition in trout’s fillets have been extensively reviewed, even if several biotic and abiotic factors contribute to the end-coloration, from strain and sex to water parameters, as pigment source and level of incorporation. In the present trial, the obtained results could be primarily attributed to the exposure of No-PAP-fed trout to higher vegetable and algae ingredients, naturally rich in xanthophylls as lutein and zeaxanthin, and are able to significantly increase the a* and b* indexes [47]. This outcome aligns with previous findings in trout fed diets containing dried microorganism biomass [48,49] or a high concentration of corn gluten meal [50]. In this study, trout fed the PAP diet exhibited the pale pink-grey flesh color reported as the preferred one in the literature [48]; however, the severe color shift observed in the No-PAP-fed diet and the moderate one found in fish fed the Mixed diet deserve further specific studies to understand consumer perception and marketability. In this regard, a recent study showed promising results about the possible acceptance of yellow-colored trout fillet, especially when information about the sustainability of the source of that color was given (i.e., *Arthrospira platensis* was utilized in the mentioned article) [51].

The present study’s results regarding fillet texture, protein, lipid, and energy content are strongly corroborated by Vale-Pereira et al. [20]. The research also supports the finding on color impact, noting a mild pink/orange pigmentation in fish fed their No-PAP+ diet (low in macro- and microalgae meal). This highlights the species-specific difficulty in achieving the desired fillet color in rainbow trout with alternative diets due to their sensitivity to ingredient pigments. Furthermore, consumer acceptance in the Vale-Pereira study was actually higher for the NO-PAP+ group compared to the PAP group due to better texture [20].

Observations from other species regarding body composition and sensory analysis are generally in line with the trout findings. For juvenile turbot, the whole-body composition (moisture, crude protein, ash, and energy content) did not significantly differ between fish fed traditional and experimental diets, although the PAP group had a slightly lower crude lipid content than the control group [40]. Market-sized turbot also showed no differences in carcass proximate composition or marketable fillet yield [39]. For European seabass, whole-body composition did not significantly differ across diets [41]. In the same study, sensory analysis of European seabass fillets, including consistency, smell, taste, and juice/grease separation, also showed no differences across any of the tested alternative diets compared to the commercial control.

The nutritional value, physical characteristics, and sensory properties, important for consumer acceptance, were largely maintained with alternative eco-efficient feeds. However, maintaining the preferred fillet color in rainbow trout, and possibly other species, may be affected by pigments present in specific ingredients, which may impact marketability.

### 4.3. Feed Formulation and Environmental Impact

An assessment of the carbon footprint (measured as GHG emissions in kg CO_2_ equivalent) of the control and the alternative diets used in the present study revealed a range from 1.40 kg CO_2_ eq. for the PAP diet to 2.35 kg CO_2_ eq. for the Ctrl diet (per kg of feed). When considering the impact per kg of fish produced, the PAP treatment showed the lowest value (1.09 kg CO_2_ eq./kg fish), while the Ctrl treatment remained the highest (1.79 kg CO_2_ eq./kg fish). The contribution analysis indicated that, for the Ctrl diet, the impact was largely associated with plant-based ingredients (67.9%), particularly SPC (34.4% of overall impact). Fish meal contributed to 18.7% of the overall impact in the Ctrl diet. For the No-PAP diet, the highest contributors to GHG emissions were Spirulina meal (15.7%) and SPC (10%). In the PAP diet, the largest contribution was related to the inclusion of Algae, *Schizochytrium* sp. (9.4%). This suggests that while these ingredients are alternatives to marine resources, their production processes may have significant environmental footprints.

Other studies echo the focus on diverse alternative ingredients and circular economy principles, and eventual trade-offs with carbon footprint and other impact categories. In a study with gilthead seabream [38] testing organic and eco-efficient feed formulations using a similar ingredient basket to the present study, the eco-efficient feed and, specially, the organic feeds had a higher carbon footprint (1.55 and 1.96 kg CO_2_ eq./kg of feed, respectively) compared to a Ctrl feed (1.41 kg CO_2_ eq./kg of feed). In that seabream study, organic and eco-efficient feeds had higher inclusions of by-products and side streams (land animal by-products, brewer’s yeast, microbial meal, and salmon oil), algae (*Arthrospira platensis* and *Schyzochytrium* sp.), and plant-based sources (e.g., potato protein concentrate, wheat gluten, and corn gluten meal), along with the reduced inclusion of marine ingredients (fishmeal, fish protein hydrolysate, and fish oil). Land animal by-products, single-cell meals, and plant ingredients have been identified as potential significant contributors to carbon emissions due to production methods, processing, and transport [26,33,52,53]. However, it should be noted that a higher carbon footprint may not necessarily bring a higher environmental impact, as a thorough Life Cycle Assessment (LCA) analyzes different impact categories, such as eutrophication, land use, water use, acidification, and other resource usage. Aquafeeds with higher inclusion of plant ingredients showed higher carbon footprint than the ones richer in marine ingredients, in Atlantic salmon and rainbow trout [54], as well as in European seabass and meagre [55]. For instance, the crop production stage in soybean meal and oil, often used as fish meal and fish oil alternatives, is responsible for the majority of the impact due to GHG emissions, eutrophication, and terrestrial ecotoxicity [56]. The substitution with plant ingredients may determine an environmental burden shifting due to the agronomic stage, particularly land and water use, fertilizers, and pesticides application. Therefore, the substitution of fish meal with plant ingredients, considering also the rapid growth of aquaculture, might lead to an increased demand for these products and the impacts associated [53]. The production process of land animal by-products contributed mostly to GHG emissions and abiotic resources depletion, while the farming stage is related to acidification potential and eutrophication [52]. The impact associated with the farming stage in land animal by-products is controversial, as other authors reported the largest contribution of this stage in the environmental impact [56].

Single-cell proteins and oils are widely viewed as a sustainable substitute for fish meal, fish oil, and plant-based ingredients, since their production uses by-products from the food or feed sector and can be located alongside these industries to improve overall efficiency [34]. In the case of DHA-rich algae production, the most impactful stages related to GHG emission is the primary production of substrates used for fermentation (heterotrophic production of *Schizochytrium* sp. from sugarcane) or the vegetable oil production used to mix the algae powder (in case liquid suspension is produced), while the impact of algae production and processing is limited to some impact categories such as particulate matter production and water use [34]. Also, the production of microalgae Spirulina and Chlorella might be conducted in a more sustainable way that allows for reducing their contribution to the overall impact. If spirulina production is conducted using a geothermal water source, the GHG emission decreases to 6.5 kg CO_2_ eq./kg compared to the 7.1 kg CO_2_ eq./kg considered in this study [57]. The impact is further reduced if spirulina is produced in an artisanal way but with a reduced yield [57]. Also, the impact associated with Chlorella could be reduced if the algae is produced in a mixotrophic way using by-products from cheese production as a source of C [30].

Similarly, the rearing substrate for insect larvae can have a considerable impact on insect meal production [58]. For instance, the use of municipal organic waste in insect rearing can reduce the environmental impact associated with GHG emissions and energy demand [59]. Moreover, the carbon footprint of land animal by-products, single-cell meals, and other ingredients with high energy usage tends to greatly decrease when renewable/clean energies are used, in line with societal trends for decarbonization [60,61,62]. In addition, the inclusion of ingredients with high carbon footprint in aquafeeds may, in certain circumstances, be a trade-off to improve system-wide performance to promote circular economy by valorization of locally available side streams and by-products from other industries [52,62]. At the same time, transport can determine a significant share of impact, for instance, for the carbon footprint associated with the production of fish meal and fish oil [56]. In the present study, the environmental impact was intentionally calculated, limiting the system boundaries to raw material production, to better reflect the environmental burden of the diet, while avoiding the geographical and temporal variability in transport’s impact.

## 5. Conclusions

This study demonstrates that novel, eco-efficient aquafeed formulations can achieve growth performance and quality standards comparable to traditional diets in rainbow trout. The successful maintenance of growth and feed efficiency across all treatments validates the use of a “basket-based” formulation rationale and highlights the efficacy of functional nutritional balancing—prioritizing the delivery of essential nutrients over fixed ingredient percentages. The consistency in growth metrics, fillet texture, and proximate composition across all formulations is highly promising for the adoption of more eco-efficient aquafeeds. However, this study identifies a trade-off: trout fed the No-PAP diet developed a distinct yellow tint in the fillet, likely resulting from the inclusion of microalgae and seaweed pigments. While this shift does not compromise nutritional quality, it represents a critical factor for consumer acceptance and marketability.

Furthermore, the environmental impact assessment reveals a nuanced sustainability profile. All the experimental diets showed a reduced environmental impact in GHG emissions compared to the control diet. Among the experimental diets, the PAP-based diet achieved the lowest CO_2_ equivalent emissions, while the No-PAP diet exhibited the highest carbon footprint, largely driven by the current production impacts of algae and soy protein concentrate. These findings underscore the necessity of comprehensive LCA when evaluating the holistic sustainability of alternative ingredients.

In conclusion, this research supports the broader shift towards reducing reliance on traditional marine and terrestrial resources and fostering a more circular economy in aquaculture, demonstrating that eco-efficient aquafeeds can maintain high standards in growth and quality for rainbow trout, provided that sensory and environmental trade-offs are further optimized.

## Figures and Tables

**Table 1:** Formulation and proximate composition of experimental diets (DM: Dry Matter).

Ingredients	Ctrl	No-PAP	PAP	Mix
	(% DM)			
Fishmeal LT701	20.0	5.0	5.0	-
Fish hydrolysate (by-products)	3.0	3.0	3.0	3.0
Insect meal (*Hermetia illucens*)	-	5.0	5.0	10.0
Microbial protein meal	-	5.0	5.0	10.0
Yeast protein meal	-	3.0	3.0	3.0
Feather meal hydrolysate	-	-	5.0	5.0
Porcine hemoglobin	-	-	2.5	2.5
Poultry meal 65	-	-	20.0	10.0
Microalgae meal (*Spirulina* sp.)	-	5.0	-	5.0
Microalgae meal (*Chlorella* sp.)	-	0.5	-	0.5
Pea protein concentrate	-	6.0	-	-
Wheat gluten	8.0	8.5	-	-
Corn gluten meal	5.0	5.0	5.0	4.5
Soy protein concentrate	18.0	5.0	-	-
Soybean meal 48	5.0	-	-	-
Wheat meal	10.0	9.25	11.95	9.75
Pea starch	5.0	5.0	5.0	5.0
Fish oil	7.4	3.7	3.7	3.7
Salmon oil (by-products)	-	8.0	8.0	8.0
DHA-rich algae (*Schizochytrium* sp.)	-	3.2	3.2	3.2
Rapeseed oil	9.7	2.8	-	0.6
Linseed oil	4.1	4.1	4.1	4.1
Rapeseed lecithin	0.5	1.0	1.0	1.0
Vitamin and mineral premix	1.0	1.0	1.0	1.0
Vitamin C (35%)	0.1	0.1	0.1	0.1
Betaine HCl	0.28	0.28	0.28	0.28
Brewer’s yeast	-	4.0	4.0	4.0
Macroalgae Mix	-	1.0	1.0	1.0
Antioxidant	0.35	0.35	0.35	0.35
Sodium propionate	0.1	0.1	0.1	0.1
Monocalcium phosphate	1.9	2.85	1.3	2.2
L-Lysine	0.3	1.0	0.5	0.95
L-Tryptophan	0.1	0.3	0.2	0.25
DL-Methionine	0.15	0.55	0.4	0.6
L-Taurine	-	0.4	0.3	0.3
Yttrium oxide	0.02	0.02	0.02	0.02
**Proximate composition (% DM)**				
Crude protein	44.91	46.05	44.46	46.76
Crude fat	24.54	20.36	24.41	19.67
Ash	7.80	6.59	6.21	6.17
Energy (kJ/g)	23.53	23.40	24.08	23.71

**Table 2:** Environmental impact (greenhouse gas emission as kg CO_2_ eq.) of the ingredients used in feed formulation.

Ingredients	kg CO_2_ eq./kg Ingredient	Description	Reference
Fishmeal LT70	2.20	Fish meal, FF LT Supreme, Skagen Denmark, 70% CP	[24]
Fish hydrolysate (by-products)	1.85	Fish hydrolysate (CPSP), Chile, at plant	[25]
Insect meal ^a^	1.74	*Hermetia illucens* larvae meal	[26]
Microbial protein meal	2.23	FeedKind, Calysta	[27]
Yeast protein meal	2.10	*Saccharomyces cerevisiae* ACTISAF SC 47	[28]
Feather meal hydrolysate	0.76	Feather meal, steam hydrolyzed	[24]
Porcine hemoglobin	0.90	Hemoglobin powder, 92% CP, SONAC	[24]
Poultry meal 65	0.45	Transformed animal proteins, from broiler, France, at plant	[25]
Microalgae meal (*Spirulina* sp.) ^b^	7.10	Spirulina powder	[29]
Microalgae meal (*Chlorella* sp.) ^c^	3.07	Chlorella powder from autotrophic production	[30]
Pea protein concentrate ^d^	1.91	Pea protein concentrate at 46.0% protein	[31]
Wheat gluten	2.60	Wheat gluten meal, from wheat starch extraction, France, at plant	[25]
Corn gluten meal	1.18	Corn gluten meal (gluten 60), national average, France, at plant	[25]
Soy protein concentrate	4.49	Soy protein concentrate, 70% CP	[24]
Soybean meal 48	2.45	Soybean meal, dehulled, 48% CP, solvent extracted	[24]
Wheat meal	0.78	Wheat, flour	[24]
Pea starch	0.85	Pea starch powder	[32]
Fish oil	1.26	Average whole fish oil	[33]
Salmon oil (by-products)	0.71	Fish oil, Atlantic salmon, farmed by product	[24]
DHA-rich algae (*Schizochytrium* sp.)	4.12	Algae omega-3 DHA liquid suspension from heterotrophically grown microalgae	[34]
Rapeseed oil	1.86	Rapeseed oil, crude, France, at plant	[25]
Linseed oil	2.12	Flaxseed oil, France, at plant	[25]
Rapeseed lecithin ^e^	2.44	Rapeseed lecithin, Europe	[35]
Vitamin and mineral premix	0.89	Vitamin premix IAFFD Standard, FW fish grower, 0.5%	[24]
Vitamin C (35%) ^f^	1.00	Rovimix-stay-C 35, ascorbyl-monophosphate, DSM	[24]
Betaine HCl ^f^	5.00	Betaine	[24]
Brewer’s yeast	2.10	*Saccharomyces cerevisiae* ACTISAF SC 47	[28]
Macroalgae Mix	0.10	Optimized seaweed (*Laminaria*) production, France	[36]
Antioxidant ^f^	20.00	BHA	[24]
Sodium propionate ^f^	20.00	Mold inhibitor (calcium propionate)	[24]
Monocalcium phosphate	1.12	Monocalcium phosphate, Europe, at plant	[25]
L-Lysine	2.37	L-Lysine HCl, France, at plant	[25]
L-Tryptophan ^f^	4.75	L-tryptophane, France, at plant	[25]
DL-Methionine ^f^	3.12	DL-methionine, Europe, at plant	[25]
L-Taurine ^f^	6.00	L-Taurine	[24]
Yttrium oxide ^f^	75.00	Yttrium oxide	[24]

Legend: ^a^ Average value Scenario 1 and 2; ^b^ Excluding tablet production; ^c^ Scenario 2 and 3 considered; ^d^ only impacts associated with sub-system 1 (pea protein concentrate production); ^e^ reference not found; ^f^ Less than 1% of inclusion in all the treatments.

**Table 3:** Texture characteristics of trout fillets at the end of the experiment. Values are expressed as mean ± SE.

Texture	Ctrl ^i^	No-PAP ^ii^	PAP ^iii^	Mix ^iv^
Hardness (N)	4.95 ± 0.29	4.89 ± 0.23	5.38 ± 0.21	4.83 ± 0.15
Cohesiveness	0.21 ± 0.007	0.21 ± 0.004	0.19 ± 0.005	0.20 ± 0.002
Resilience (N*mm)	0.02 ± 0.003	0.03 ± 0.002	0.03 ± 0.003	0.02 ± 0.002
Gumminess (N)	0.99 ± 0.05	1.00 ± 0.04	1.05 ± 0.05	0.95 ± 0.04
Adhesiveness (N*mm)	0.60 ± 0.05	0.50 ± 0.03	0.54 ± 0.04	0.61 ± 0.03

^i^ Control diet (Ctrl); ^ii^ diet without Processed Animal Proteins (No-PAP); ^iii^ diet with Processed Animal Proteins (PAP); and ^iv^ mixed diet (Mix).

**Table 4:** Table presenting mean ± SE values of color indices per diet, including lightness (L*), redness (a*), yellowness (b*), chroma (C), hue (h), and the entire color index (ECI). Different superscript letters indicate statistically significant differences (*p* < 0.05) between groups.

	Ctrl ^i^	No-PAP ^ii^	PAP ^iii^	Mix ^iv^
L*	43.94 ± 0.17 ^b^	42.61 ± 0.14 ^c^	45.14 ± 0.16 ^a^	43.76 ± 0.14 ^b^
a*	2.69 ± 0.16 ^c^	4.35 ± 0.15 ^a^	3.13 ± 0.16 ^bc^	3.46 ± 0.15 ^b^
b*	9.19 ± 0.22 ^b^	15.46 ± 0.31 ^a^	5.26 ± 0.18 ^c^	9.42 ± 0.24 ^b^
C	9.69 ± 0.26 ^b^	16.12 ± 0.33 ^a^	6.24 ± 0.22 ^c^	10.13 ± 0.26 ^b^
h	76.04 ± 0.60 ^a^	74.88 ± 0.33 ^a^	61.17 ± 0.79 ^c^	70.64 ± 0.61 ^b^
ECI	5.03 ± 0.28 ^c^	8.37 ± 0.27 ^a^	5.32 ± 0.23 ^c^	6.50 ± 0.25 ^b^

^i^ Control diet (Ctrl), ^ii^ diet without Processed Animal Proteins (No-PAP), ^iii^ diet with Processed Animal Proteins (PAP), and ^iv^ mixed diet (Mix); *n* = 216 reads per group (24 fish with 3 fillet-areas each and 3 reads per area).

**Table 5:** Fillet composition (fresh matter; mean ± SD) of fish fed different experimental diets at the beginning (Initial reference) and end of the trial.

	Ctrl ^i^	No-PAP ^ii^	PAP ^iii^	Mix ^iv^	Initial Reference
Moisture (%)	66.46 ± 0.44	65.23 ± 0.19	65.54 ± 1.22	65.90 ± 0.60	76.45
Ash (%)	1.57 ± 0.23	1.57 ± 0.23	1.55 ± 0.17	1.55 ± 0.21	1.47
Protein (%)	16.27 ± 0.29	16.62 ± 0.31	16.24 ± 0.05	16.15 ± 0.66	15.92
Fat (%)	12.90 ± 1.10	11.24 ± 3.09	12.38 ± 2.21	13.93 ± 2.48	5.37
Energy (kJ/g)	9.10 ± 0.30	9.65 ± 0.16	10.42 ± 1.54	9.35 ± 0.17	5.77

^i^ Control diet (Ctrl), ^ii^ diet without Processed Animal Proteins (No-PAP), ^iii^ diet with Processed Animal Proteins (PAP), and ^iv^ mixed diet (Mix).

**Table 6:** Relative contribution of each ingredient (%) on the overall impact of diets (kg CO_2_ eq./kg feed) and fish produced (kg CO_2_ eq./kg fish), considering the feed conversion rate obtained in the present trial.

Ingredients	Ctrl ^i^	No-PAP ^ii^	PAP ^iii^	Mix ^iv^
Feed conversion rate	0.76	0.78	0.78	0.79
**Overall impact as kg CO_2_ eq.**				
Overall impact per kg of feed	2.35	2.25	1.40	1.83
Overall impact per kg of fish	1.79	1.76	1.09	1.45
**Relative contribution of each ingredient (%)**				
Fishmeal LT701	18.7%	4.9%	7.9%	
Fish hydrolysate (by-products)	2.4%	2.5%	4.0%	3.0%
Insect meal (*Hermetia illucens*)		3.9%	6.2%	9.5%
Microbial protein meal		4.9%	8.0%	12.2%
Yeast protein meal		2.8%	4.5%	3.4%
Feather meal hydrolysate			2.7%	2.1%
Porcine hemoglobin			1.6%	1.2%
Poultry meal 65			6.4%	2.4%
Microalgae meal (*Spirulina* sp.)		15.7%		19.4%
Microalgae meal (*Chlorella* sp.)		0.7%		0.8%
Pea protein concentrate		5.1%		
Wheat gluten	8.8%	9.8%		
Corn gluten meal	2.5%	2.6%	4.2%	2.9%
Soy protein concentrate	34.4%	10.0%		
Soybean meal 48	5.2%			
Wheat meal	3.3%	3.2%	6.6%	4.1%
Pea starch	1.8%	1.9%	3.0%	2.3%
Fish oil	4.0%	2.1%	3.3%	2.5%
Salmon oil (by-products)		2.5%	4.1%	3.1%
DHA-rich algae (*Schizochytrium* sp.)		5.8%	9.4%	7.2%
Rapeseed oil	7.6%	2.3%		0.6%
Linseed oil	3.7%	3.9%	6.2%	4.8%
Rapeseed lecithin	0.5%	1.1%	1.7%	1.3%
Vitamin and mineral premix	0.4%	0.4%	0.6%	0.5%
Vitamin C (35%)	0.0%	0.0%	0.1%	0.1%
Betaine HCl	0.6%	0.6%	1.0%	0.8%
Brewer’s yeast		3.7%	6.0%	4.6%
Macroalgae Mix		0.0%	0.1%	0.1%
Antioxidant	3.0%	3.1%	5.0%	3.8%
Sodium propionate	0.9%	0.9%	1.4%	1.1%
Monocalcium phosphate	0.9%	1.4%	1.0%	1.3%
L-Lysine	0.3%	1.1%	0.8%	1.2%
L-Tryptophan	0.2%	0.6%	0.7%	0.6%
DL-Methionine	0.2%	0.8%	0.9%	1.0%
L-Taurine		1.1%	1.3%	1.0%
Yttrium oxide	0.6%	0.7%	1.1%	0.8%

^i^ Control diet (Ctrl), ^ii^ diet without Processed Animal Proteins (No-PAP), ^iii^ diet with Processed Animal Proteins (PAP), and ^iv^ mixed diet (Mix).

## Data Availability

The original contributions presented in this study are included in the article/Supplementary Material. Further inquiries can be directed to the corresponding authors.

## Supplementary Materials

The following supporting information can be downloaded at: https://www.mdpi.com/article/10.3390/ani16071000/s1, File S1: Supplementary Materials; Figure S1: Water temperature (T) and dissolved oxygen (DO) in experimental tanks during the trial; Table S1: Formulation and proximate composition of the experimental diets for Trout; Table S2: Dietary amino acid content for Trout; Table S3: Fatty acid content of the diets; Table S4: Mineral composition of the diets; Table S5: Vitamin content of the diets; Table S6: Ingredients details. References [63,64,65] are cited in the Supplementary Materials.

## Abbreviations

The following abbreviations are used in this manuscript:

Abbreviation	Full Name
a*	Redness Index (CIE-Lab Coordinate)
ADC	Apparent Digestibility Coefficient
ANOVA	Analysis of Variance
AOAC	Association of Official Analytical Chemists
b*	Yellowness Index (CIE-Lab Coordinate)
BW	Body Weight
C	Chroma (Color Intensity)
CO_2_ eq.	Carbon Dioxide Equivalent
CPI	Crude Protein Intake
Ctrl	Control Diet
CY	Carcass Yield
DHA	Docosahexaenoic Acid
DM	Dry Matter
ECI	Entire Color Index
FAO	Food and Agriculture Organization
FBW	Final Body Weight
FCR	Feed Conversion Ratio
FI	Feed Intake
FM	Fishmeal
FO	Fish Oil
GHG	Greenhouse Gas
h	Hue (Color Angle)
$\bar{h}$	Mean Hue
HSI	Hepato-somatic Index
IBW	Initial Body Weight
L*	Lightness (CIE-Lab Coordinate)
LCA	Life Cycle Assessment
MFI	Mesenteric Fat Index
MD	Marker Diet
MF	Marker Feces
Mix	Mixed Diet
No-PAP	Diet Without Processed Animal Proteins
PAP	Processed Animal Proteins
PER	Protein Efficiency Ratio
SGR	Specific Growth Rate
SD	Standard Deviation
SPC	Soy Protein Concentrate
TND	Target Nutrient Diet
TNF	Target Nutrient Feces
TPA	Texture Profile Analysis
VSI	Viscero-somatic Index
WG	Weight Gain
Y_2_O_3_	Yttrium Oxide
