# Supplementary material for: Practical Aquafeeds Incorporating Insect and Algae Meals Achieve Quality and Growth Standards Comparable to Traditional Feeds in Rainbow Trout (Oncorhynchus mykiss)
Source: Animals (Basel). 2026 Mar 24;16(7):1000. doi: 10.3390/ani16071000 (PMC13071961; doi:10.3390/ani16071000)
Supplement: Supplementary file 1 [file animals-16-01000-s001.zip › animals-4048663-supplementary.pdf]

Manufacture of experimental diets

Diets were formulated according to the known nutritional requirements for Rainbow trout (NRC 2011) and manufactured by extrusion at SPAROS facilities. All powder ingredients were admixed accordingly to the target formulation in a double-helix mixer (model 500L, TGC Extrusion Roullet Saint Estèphe, France) and ground (below 400 μm) in a micro pulveriser hammer mill (model SH1, Hosokawa-Alpine AG, Augsburg, Germany). Diets (pellet size changed according to species and fish size) were manufactured with a twin-screw extruder (model BC45, Clextral, Firminy, France) with a screw diameter of 55.5 mm. Extrusion conditions: feeder rate (80-85 kg/h), screw speed (247-266 rpm), water addition in barrel 1 (345 ml/min), temperature barrel 1 (32-34ºC), temperature barrel 3 (111-117°C). Extruded pellets were dried in a vibrating fluid bed dryer (model DR100, TGC Extrusion, Roullet Saint Estèphe, France). After cooling, an oils blend was embedded by vacuum coating (model PG-10VCLAB, Dinnissen DV, Sevenum, The Netherlands). Coating conditions were: pressure (700 mbar); spraying time under vacuum (approximately 90 seconds), return to atmospheric pressure (120 seconds). Immediately after coating, diets were packed in bags or sealed plastic buckets and shipped to the research site where they were stored at room temperature, but in a cool and aerated emplacement. Representative amounts of each diet were collected for composition analysis (Table S1).

Analytic

For fatty acid analysis of the feeds, lipids were extracted following the method of Folch et al. (1957) [1], and fatty acid composition was analyzed through gas chromatography of methyl esters, based on the procedure described by Lepage and Roy (1986) [2]. For mineral analysis, dry samples weighing between 50 and 200 mg were placed in quartz vessels and digested in 6 mL of 70% nitric acid (HNO_3, tracer grade) using a Discovery SP-D microwave digestion unit. The digestion program involved a ramp to 200 ℃ over 4 minutes, followed by a 3-minute hold. After cooling to room temperature, ultrapure water was added to achieve a final volume of 10 mL. Samples were then diluted 16 times with ultrapure water, and standard curves were created in ultrapure water. Mineral quantification was conducted using MP-AES (model 4200, Agilent Technologies Inc., Santa Clara, CA, USA). Blank samples containing only the decomposition acid were included to account for matrix effects, which were subtracted from each element's measurements in the samples.

Table S1: Formulation and proximate composition of the experimental diets for Trout.

**Ingredients, %**	**Ctrl**	**No-PAP**	**PAP**	**Mix**
Fishmeal LT70	20.000	5.000	5.000	
Fish hydrolysate (by-products)	3.000	3.000	3.000	3.000
Insect meal		5.000	5.000	10.000
Microbial protein meal		5.000	5.000	10.000
Yeast protein meal		3.000	3.000	3.000
Feather meal hydrolysate			5.000	5.000
Porcine hemoglobin			2.500	2.500
Poultry meal 65			20.000	10.000
Microalgae meal (*Spirulina*)		5.000		5.000
Microalgae meal (*Chlorella*)		0.500		0.500
Pea protein concentrate		6.000		
Wheat gluten	8.000	8.500		
Corn gluten meal	5.000	5.000	5.000	4.500
Soy protein concentrate	18.000	5.000		
Soybean meal 48	5.000			
Wheat meal	10.000	9.250	11.950	9.750
Pea starch	5.000	5.000	5.000	5.000
Fish oil	7.400	3.700	3.700	3.700
Salmon oil (by-products)		8.000	8.000	8.000
DHA-rich algae (*Schizochytrium*)		3.200	3.200	3.200
Rapeseed oil	9.700	2.800		0.600
Linseed oil	4.100	4.100	4.100	4.100
Rapeseed lecithin	0.500	1.000	1.000	1.000
Vitamin and mineral premix	1.000	1.000	1.000	1.000
Vitamin C (35%)	0.100	0.100	0.100	0.100
Betaine HCl	0.280	0.280	0.280	0.280
Brewer's yeast		4.000	4.000	4.000
Macroalgae mix		1.000	1.000	1.000
Antioxidant	0.350	0.350	0.350	0.350
Sodium propionate	0.100	0.100	0.100	0.100
Monocalcium phosphate	1.900	2.850	1.300	2.200
L-Lysine	0.300	1.000	0.500	0.950
L-Tryptophan	0.100	0.300	0.200	0.250
DL-Methionine	0.150	0.550	0.400	0.600
L-Taurine		0.400	0.300	0.300
Yttrium oxide	0.020	0.020	0.020	0.020
**Total**	**100.00**	**100.00**	**100.00**	**100.00**
**Feed composition (%DM)**	**Ctrl**	**No-PAP**	**PAP**	**Mix**
Crude protein	44.91	46.05	44.46	46.76
Crude fat	24.54	20.36	24.41	19.67
Energy (kJ/g)	23.53	23.40	24.08	23.71
Ash	7.80	6.59	6.21	6.17

Table S2: Dietary amino acid content for Trout. Values are mean and standard deviation of duplicates analysis (*n*=2).

Amino acids (%DM)	Ctrl		No-PAP		PAP		Mix		Trout requirement [3]
	Mean	SD	Mean	SD	Mean	SD	Mean	SD	
**Arginine (Arg)**	2.92	0.18	2.80	0.15	2.74	0.07	2.82	0.03	**1.74**
**Histidine (His)**	1.17	0.09	1.05	0.03	0.99	0.03	1.12	0.10	**0.83**
**Isoleucine (Ile)**	1.81	0.01	1.80	0.09	1.73	0.09	1.74	0.05	**1.16**
**Leucine (Leu)**	3.14	0.04	3.14	0.21	3.18	0.10	3.19	0.14	**2.16**
**Lysine (Lys)**	2.83	0.04	2.97	0.05	2.80	0.22	2.92	0.10	**2.29**
**Threonine (Thr)**	1.83	0.02	1.69	0.04	1.61	0.09	1.74	0.03	**1.44**
**Tryptophan (Trp)***	0.48	-	0.64	-	0.52	-	0.68	-	**0.33**
**Valine (Val)**	1.91	0.01	1.85	0.03	2.00	0.09	2.16	0.02	**1.48**
**Methionine (Met)**	1.13	0.03	1.20	0.02	1.12	0.09	1.18	0.03	**0.84**
**Cystine (Cys)**	0.33	0.01	0.28	0.00	0.29	0.00	0.31	0.01	-
**Phenylalanine (Phe)**	2.43	0.18	2.31	0.03	2.26	0.19	2.15	0.02	**1.18**
**Tyrosine (Tyr)**	1.84	0.15	1.91	0.05	1.53	0.00	1.80	0.04	-
**Aspartic acid + Asparagine (Asx)**	3.41	0.02	2.93	0.05	3.25	0.30	3.02	0.18	-
**Glutamic acid + Glutamine (Glx)**	7.78	0.35	7.22	0.23	6.65	0.39	6.78	0.20	-
**Alanine (Ala)**	2.03	0.08	1.97	0.12	2.23	0.02	2.31	0.18	-
**Glycine (Gly)**	1.78	0.12	1.70	0.12	2.15	0.11	2.05	0.06	-
**Proline (Pro)**	2.68	0.01	2.64	0.18	2.89	0.04	2.67	0.20	-
**Serine (Ser)**	2.17	0.01	1.82	0.04	1.91	0.06	1.90	0.07	-
**Taurine (Tau)**	0.46	0.02	0.71	0.04	0.60	0.04	0.53	0.02	-

* Value not made in duplicate, thus no standard deviation calculated.

Table S3: Fatty acid content of the diets.

Fatty acids	Ctrl	No-PAP		PAP		Mix		Trout requirement [3]
	*	Mean	SD	Mean	SD	Mean	SD	
**14:0**	0.270	0.251	0.004	0.319	0.037	0.272	0.018	-
**15:0**	0.000	0.000	0.000	0.000	0.000	0.000	0.000	-
**16:0**	1.563	1.587	0.003	1.995	0.059	2.071	0.134	-
**18:0**	0.367	0.357	0.001	0.448	0.002	0.489	0.033	-
**20:0**	0.000	0.000	0.000	0.000	0.000	0.000	0.000	-
**22:0**	0.000	0.000	0.000	0.000	0.000	0.000	0.000	-
**24:0**	0.027	0.024	0.008	0.021	0.001	0.014	0.020	-
**Total saturated**	2.226	2.219	0.007	2.784	0.100	2.846	0.129	-
**16:1**	0.446	0.470	0.014	0.583	0.025	0.666	0.047	-
**18:1n-9**	6.024	5.001	0.131	5.768	0.046	6.364	0.168	-
**18:1n-7**	0.000	0.000	0.000	0.000	0.000	0.000	0.000	-
**20:1**	0.232	0.296	0.022	0.349	0.010	0.392	0.022	-
**22:1**	0.000	0.000	0.000	0.000	0.000	0.000	0.000	-
**24:1**	0.027	0.030	0.000	0.030	0.001	0.034	0.002	-
**Total monounsaturated**	6.730	5.798	0.140	6.730	0.032	7.456	0.239	-
**18:2n-6**	2.698	2.439	0.073	2.644	0.039	2.528	0.061	-

**18:3n-6**	0.000	0.040	0.009	0.011	0.016	0.036	0.006	-
**20:2n-6**	0.000	0.000	0.000	0.000	0.000	0.000	0.000	-
**20:3n-6**	0.000	0.000	0.000	0.000	0.000	0.000	0.000	-
**20:4n-6**	0.045	0.031	0.001	0.043	0.003	0.044	0.004	-
**22:4n-6**	0.000	0.000	0.000	0.000	0.000	0.000	0.000	-
**22:5n-6**	0.000	0.000	0.000	0.000	0.000	0.000	0.000	-
**Total n-6 PUFA**	2.742	2.510	0.083	2.698	0.026	2.608	0.071	**0.4**
**18:3n-3**	1.393	1.241	0.108	1.409	0.060	1.594	0.062	-
**18:4n-3**	0.087	0.066	0.008	0.077	0.000	0.078	0.007	-
**20:3n-3**	0.000	0.000	0.000	0.000	0.000	0.000	0.000	-
**20:4n-3**	0.000	0.000	0.000	0.000	0.000	0.000	0.000	-
**20:5n-3**	1.011	0.640	0.023	0.720	0.048	0.803	0.096	-
**21:5n-3**	0.000	0.000	0.000	0.000	0.000	0.000	0.000	-
**22:4n-3**	0.000	0.000	0.000	0.000	0.000	0.000	0.000	-
**22:5n-3**	0.105	0.117	0.004	0.145	0.003	0.161	0.007	-
**22:6n-3**	0.639	0.579	0.032	0.606	0.010	0.601	0.052	-
**Total n-3 PUFA**	3.235	2.644	0.065	2.957	0.001	3.237	0.224	**0.96**
**Total PUFA**	5.978	5.154	0.018	5.655	0.025	5.845	0.295	-
**Total FA**	14.93	13.325	0.076	15.337	0.070	16.330	0.683	-

* Due to technical problems with the machine control diets are shown without replicate and no standard deviation was calculated.

Table S4: Mineral composition of the diets. Values are mean and standard deviation of technical replicates (*n*=2).

**Minerals**	**Ctrl**		**No-PAP**		**PAP**		**Mix**	
	**Mean**	**SD**	**Mean**	**SD**	**Mean**	**SD**	**Mean**	**SD**
**% DM**								
**P**	1.30	0.01	1.33	0.04	1.11	0.02	1.22	0.00
**Ca**	1.58	0.01	1.17	0.03	1.38	0.03	1.11	0.00
**Na**	0.48	0.01	0.48	0.01	0.42	0.01	0.39	0.00
**Mg**	0.18	0.00	0.16	0.00	<LOQ	-	0.16*	-
**K**	0.95	0.02	0.53	0.01	0.42	0.01	0.44	0.00
**mg/kg**								
**As**	3.99	0.57	1.94	0.10	1.85	0.19	1.71	0.09
**Cu**	13.54	0.19	25.09	0.59	24.74	0.27	34.21	0.23
**Fe**	177.04	0.87	329.84	3.48	331.16	2.84	410.69	4.17
**Mn**	69.15	13.39	104.16	37.60	82.16	2.14	86.04	12.30
**Y**	167.10	4.55	169.62	5.40	176.01	1.95	172.93	2.47
**Zn**	187.12	8.28	197.74	24.73	197.79	10.69	197.15	5.20

<LOQ means values were too low for quantification. *Second duplicate was too low for quantification; thus, no standard deviation was calculated.

Table S5: Vitamin content of the diets.

**Vitamins**	**Ctrl**	**No-PAP**	**PAP**	**Mix**
**mg/100g**				
Vit E	25.00	25.9	22.5	19.8
Vit B1	0.244	0.22	0.237	0.219
Vit B2	0.81	0.914	0.939	0.88
Vit B3	5.64	6.6	18.2	14.1
Vit B6	0.491	0.516	0.466	0.533

**mg/kg**				
Vit B5 mg/kg	62.6	61.1	62.2	60.9
Vit C mg/kg	477	482	467	546
**ug/100g**				
Vit B9	461	534	546	541
Vit B12	36.9	96.3	96.2	118

Table S6: Ingredients details.

**Ingredients**	**Details**
Fishmeal LT70	NORVIK LT, Sopropêche: CP 71.9 %, CF 6.8 % (France)
Fish hydrolysate (by-products)	GAIN project custom fish byproducts hydrolysate: CP 82.6 %, CF 9.6 % (IIM-CSIC, Spain)
Insect meal	*Hermetia illucens* CP 57.8 %, CF 8.5 % (Supplier not disclosed)
Microbial protein meal	Methanotrophic bacteria, CP 68.2 %, CF 9.8 % (Supplier not disclosed)
Yeast protein meal	*Saccharomyces cerevisiae*, CP 68.0%, CF 0.9 % (Supplier not disclosed)
Feather meal hydrolysate	SONAC BV: CP 82.9 %, CF 11.2 % (The Netherlands)
Porcine hemoglobin	SONAC BV: CP 91.6%, CF 1.2 % (The Netherlands)
Poultry meal 65	SAVINOR UTS: CP 62.4 %, CF 14.5 % (Portugal)
Microalgae meal (*Spirulina sp.*)	*Arthrospira platensis*, Sopropêche: CP 72.1%, CF 1.0 % (France)
Microalgae meal (*Chlorella sp.*)	*Chlorella vulgaris*, Allmicroalgae: CP 62.5%, CF 9.2 % (Portugal)
Pea protein concentrate	Lysamine GPS, Roquette Frères: CP 78.1 %, CF 0.9% (France)
Wheat gluten	VITAL, Roquette Frères: CP 80.4%, CF 5.6% (France)
Corn gluten meal	COPAM: CP 61.2 %, CF 6.1 % (Portugal)
Soy protein concentrate	Soycomil, ADM: CP 62.2 %, CF 0.7 % (The Netherlands)
Soybean meal 48	Dehulled solvent extracted, CARGILL: CP 47.4%, CF 2.6 % (Spain)
Wheat meal	Casa Lanchinha: 11.7 %, CF 1.6 % (Portugal)
Pea starch	NASTAR, COSUCRA: 90% starch (Belgium)
Fish oil	Sopropêche: CF 98.1%, 16% EPA, 12% DHA (France)
Salmon oil (by-products)	Sopropêche: CF 98.3%, 4.6% EPA, 5.2% DHA (France)
DHA-rich algae (*Schizochytrium sp.*)	ALL-G Rich, Alltech: CF 63.0%, 16% DHA (Ireland)
Rapeseed oil	Henry Lamotte Oils GmbH: CF 98.2% (Germany)
Linseed oil	Henry Lamotte Oils GmbH: CF 98.4% (Germany)
Rapeseed lecithin	CANOLACITHIN F60: CF 94.0%, Novastell (France)
Vitamin and mineral preMix	*
Vitamin C (35%)	ROVIMIX Stay C35, DSM Nutritional Products (Switzerland)
Betaine HCl	Beta-Key 95%, ORFFA (The Netherlands)
Brewer's yeast	Z Premix Lda: CP 38.9%, CF 4.5% (Portugal)
Macroalgae Mix	OceanFeed, Ocean Harvest: CP 11%, CF 0.6% (Ireland)
Antioxidant	VERDILOX, Kemin Europe NV (Belgium)
Sodium propionate	Disproquímica (Portugal)
Monocalcium phosphate	ALIPHOS MONOCAL: 22.7% P, 17.5% Ca (Belgium)
L-Lysine	Ajinomoto EUROLYSINE S.A.S 99% Lys (France)
L-Tryptophan	Ajinomoto EUROLYSINE S.A.S 98% Trp (France)

DL-Methionine	DL-Met for Aquaculture, EVONIK Nutrition & Care GmbH: 99% Met (Germany)
L-Taurine	ORFFA: 98% Tau (The Netherlands)
Yttrium oxide	Sigma Aldrich (USA).

*In all diets, the inclusion of the vitamin and mineral premix contributed to an additional supply of the following micronutrients. Vitamins (IU or mg·kg-1 diet): DL-alpha tocopherol acetate, 255 mg; sodium menadione bisulphate, 10 mg; retinyl acetate, 26000 IU; DL-cholecalciferol, 2500 IU; thiamine, 2 mg; riboflavin, 9 mg; pyridoxine, 5 mg; cyanocobalamin, 0.5 mg; nicotinic acid, 25 mg; folic acid, 4 mg; L-ascorbic acid monophosphate, 80 mg; inositol, 17.5 mg; biotin, 0.2 mg; calcium panthotenate, 60 mg; choline chloride, 1960 mg. Minerals (g or mg·kg-1 diet): copper sulphate, 8.25 mg; ferric sulphate, 68 mg; potassium iodide, 0.7 mg; manganese oxide, 35 mg; organic selenium, 0.01 mg; zinc sulphate, 123 mg; calcium carbonate, 1.5 g; excipient wheat middlings.

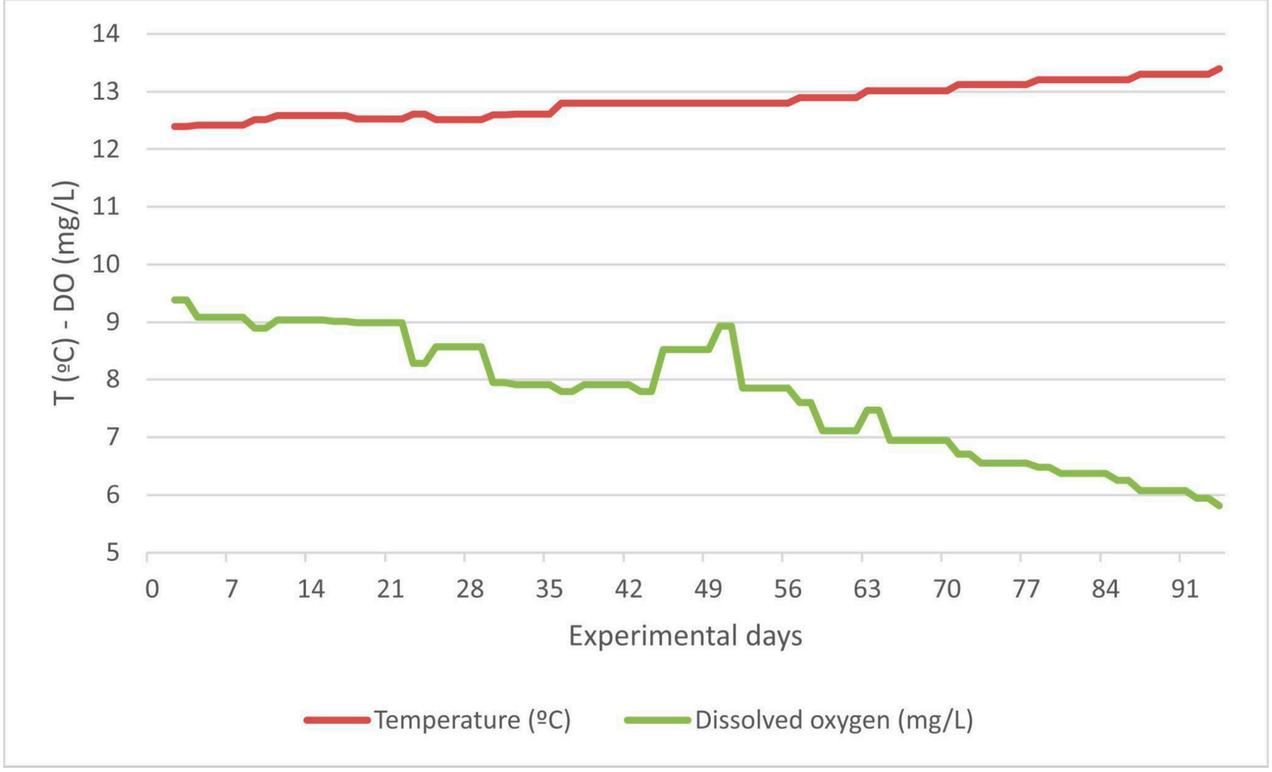

Figure S1. Water temperature (T) and dissolved oxygen (DO) in experimental tanks during the trial.

Bibliography

1. Folch, J.; Lees, M.; Stanley, G.H.S. A SIMPLE METHOD FOR THE ISOLATION AND PURIFICATION OF TOTAL LIPIDES FROM ANIMAL TISSUES. *J. Biol. Chem.* **1957**, *226*, 497–509, doi:10.1016/S0021-9258(18)64849-5.
2. Lepage, G.; Roy, C.C. Direct Transesterification of All Classes of Lipids in a One-Step Reaction. *J. Lipid Res.* **1986**, *27*, 114–120, doi:10.1016/S0022-2275(20)38861-1.
3. International Aquaculture Feed Formulation Database (IAFFD). Aquaculture Species Nutritional Specifications (ASNS), Version 11.0. Available online: https://iaffd.com/ (accessed on 27 December 2025).
